# Traditional bone setting service users and associated factors among people with trauma in Mecha district, Ethiopia

**DOI:** 10.1186/s12906-023-03951-8

**Published:** 2023-05-03

**Authors:** Belaynew Adugna Endeshaw, Worku Belay, Aderaw Gete, Kassawmar Angaw Bogale, Bekalu Wubeshet, Abebaw Gedef Azene, Gizachew Tadesse Wassie, Wubshet Aderaw, Birlew Teshome, Assefa Gebeyehu Muluneh, Berihun Assefa Demissie

**Affiliations:** 1 Department of Physiotherapy, College of Medicine and Health Science, Bahir Dar University, Bahir Dar, Ethiopia; 2 Department of Orthopedics, College of Medicine and Health Science, Bahir Dar University, Bahir Dar, Ethiopia; 3 Department of Epidemiology and Biostatistics, College of Medicine and Health Science, Bahir Dar University, Bahir Dar, Ethiopia

**Keywords:** Traditional bone setting, Traditional medicine, Indigenous knowledge, Trauma, Community, Mecha District, Ethiopia

## Abstract

World Health Organization stated that traditional medicine is an important part of health care and countries need to consider integrating it into their primary health care system. Traditional bone setting has a long history in Ethiopia and it enjoys enormous acceptance in the community. However, these methods are raw, there is no standardized training and at the same time, complications are common. Therefore, this research aimed to assess the prevalence of traditional bone setting service utilization and associated factors among people with trauma in Mecha district.

**Methods** A Community- based cross-sectional study design was employed from January 15 to February 15, 2021. A total of 836 participants were selected using a simple random sampling technique. Binary and multiple logistic regressions were employed to assess the association between the independent variables with traditional bone setting service utilization.

**Results** The prevalence of traditional bone setting service utilization was 46.05%. Factors significantly associated with TBS utilization were: Age ≥ 60 years (AOR = 0.13, 95% CI: 0.03- 0.43), rural residence (AOR = 3.63, 95% CI: 1.76 -7.50), occupation (merchant (AOR = 0.21, 95% CI: 0.07 -0.61), and housewife (AOR = 4.12, 95% CI: 1.33 -12.70), type of trauma: dislocation (AOR = 6.40, 95% CI: 3.69–11.10), and strain (AOR = 2.09, 95% CI: 1.05- 4.14)), site of trauma: extremity (AOR = 0.20, 95% CI: 0.11, 0.37), trunk (AOR = 0.08, 95% CI: 0.03–0.22), and shoulder (AOR = 0.20, 95% CI: 0.11–0.37), cause of trauma: fall down and natural deformity (AOR = 9.87, 95% CI: 5.93–16.42) and household annual income greater than > 36,500 (AOR = 2.33, 95% CI: 1.29–4.22).

**Conclusion** The prevalence of traditional bone setting practice is high in the study area, despite recent advancements in the practice of orthopedics and trauma in Ethiopia. Since TBS services are more accepted in society, the integration of TBS into the health care delivery system is recommended.

## Background

According to the World Health Organization (WHO), Traditional Medicine(TM) refers to the knowledge, skills and practices based on the theories, beliefs and experiences of different indigenous cultures used in the maintenance of health which is characterized by the prevention, diagnosis, improvement or treatment of physical and mental illness [1].

Traditional Bone Setting (TBS) practice; is commonly utilized TM and there is a widespread belief in Ethiopian society that TBS is better in trauma management than modern practitioners. In Ethiopia, reports show that 65.4% of the patients had positive attitude towards the efficacy of TM [2].

It has been popular for hundreds of years and remains so in spite of the current advancement in modern medicine. The TBS practitioners (with their own strengths and weaknesses), too, have been doing a very great job for the past thousand years in Ethiopia which needs to be explored [3].

Nonetheless, TBS has several limitations. One, the practices are not scientifically based; two, the outcome of their intervention in trauma care frequently leads to quite common profound complications like loss of limbs (gangrene), lifelong deformities (nonunion, mal-union, joint stiffness and contractures), and infections of limbs, osteomyelitis and sometimes death. This is the reality in our day to day clinical practice for most of our patients who visited TBS before they come to the hospitals as shown in the next pictures [4, 5].

Majority of these morbidities are caused by the methods used in managing these fractures. Such methods include the use of bamboo, rattan cane and palm stick wrapped round fracture segments with consequent tourniquet effect. There is also the use of herbs like incantations and scarifications which result in infection and osteomyelitis [6]. These could be due to lack of formal training of bonesetters, lack of knowledge, fear of loss of source of income and ignorance about causes and effects of complications. Bone setters often do not appreciate the dangers of tight splint that can cause gangrene [7].

It is quite devastating considering most patients with such trauma are children and young people who are at productive age. Such patients end up having limb amputations and become disabled at this age.

Even though we observed large number of complicated cases in the Hospital and at community level, it lacks a denominator. We didn’t have evidence that can tell us about the magnitude of complicated case. On the other hand, no one can deny that, bonesetters have bad outcome as well as some reputable life and limb saving outcomes. But we failed to find a thorough study where we can refer the success and failure rate of this intervention.

The prevalence of complications after TBS treatment varies from 20.60% [8] in England to 52.3% in Nigeria [9]. Despite these complications, there is a great demand for TBS. Some patients prefer to leave modern hospitals in favor of treatment by a TBS [10]. In spite of recent advancements in the practice of orthopedics and trauma, there is still a very high utilization of TBS regardless of the availability and accessibility of modern healthcare services [11].

With the growing rate of accident of different forms in Ethiopia (including civil war and internal displacement) and because of fractures management malpractices; complications of fractures treatment increased, which adds a big burden on the health care system at large and the individual victims in particular [3] The affected individuals and families are posing to physical, economic and social burden, which ultimately affects the society [12].

In our community, bonesetters are one of the health care choses and they are frequently visited, easily accessible and trusted. However, in our clinical observations, we have seen that simple fractures and subluxations were changed to limb and life-threatening condition after TBS visit. Socio-cultural, economical and health care related factors associated with use of TBS treatment are still not well studied which makes challenging to reduce the debilitating effect of the complication of TBS practices.

Consequently, undertaking studies to better understand the outcome of TBS is very crucial to reduce the debilitating effect of this complication and to develop a cohesive and integrative approach to health care. Therefore, this study was intended to determine the prevalence of traditional bone setting utilization and to identify contributing factors.

## Methods and materials

### Study design and setting

Community-based cross-sectional study was employed. The study was conducted in Mecha district, Amhara National Regional State, Ethiopia. Mecha district is situated at 500 km northwest of Addis Ababa, the capital of Ethiopia, and 35 km to the west of Bahir Dar, the capital of Amhara region. The total population of Mecha district is estimated to be 375,716. It has 40 rural and 3 urban Kebeles with a total of one primary hospital, ten health centers, and forty health posts.

### Participants

The source population was all adults living in Mecha district at least for six months. Selected individuals from households with history of injury in the last one year living in Mecha district were the study population.

### Variables of the study

#### Dependent variable

Traditional bone setting utilization (Yes = 1, No = 0).

#### Independent variables

##### Socio-demographic related variables

Age, sex, residence, religion, educational status, marital status, occupation, Community based health insurance and annual income.

##### Trauma related variables

Type of trauma, sit of trauma, cause of trauma, and time since trauma.

##### Health care related variables

Distance to TBS and Hospital sites, Reason for preference, types of intervention and cost of services.

### Operational definition

#### Traditional bone setting utilization

Is patients who visit TBS only and first TBS then Hospital after having sustained trauma.

### Sample size and selection procedures

The sample size was calculated based on the following assumptions using Epi Info version 7: TBS user prevalence assumption of 0.52 taken from the previous study [13], at 95% confidence interval (CI), a design effect of 2 and non-response rate of 10%, the required sample size was 844 individuals.

The study participants were chosen using multi-stage cluster sampling technique. Assuming that injuries are random at *Kebele* level; since the district’s source of income is mainly agriculture which is prone to injury first, the district was clustered into urban and rural *Kebeles* (the smallest administrative unit in Ethiopia). Then, we selected one urban (*Kebele* 01) and ten rural *Kebeles, namely* *:* *woteteber,* Z/hiwot, awota, kurte bahir, goragot, ambomesek, dagi, dagi zuria felegehiwot and birhan chora, by simple random sampling (lottery) method. The numbers of participants in each K *ebele* were allocated proportionally after surveying households with injury. List of all households were obtained from selected *Kebeles*. Then the households were selected using simple random sampling technique. From the selected households only one individual with injury experience was selected and interviewed.

### Data collection procedure

The data was collected by interviewer administered semi structured questionnaire. Households with an individual had history of injury in the last one year was eligible for interview. Oral informed consent was taken from each participant. Four BSc nurses after trained for half a day collected the data.

### Data analysis

The collected data was coded, entered and cleaned using EPI info version 7 and then, exported and analyzed using Stata version 14. Descriptive statistics including frequency and percentage were used to describe the findings.

Inferential statistical analysis using multivariable logistic regression was employed to show the relationship between dependent and independent variables. Model goodness of fitness was checked using Hosmur and Lemishow test and its p value was 0.06. Multivariable logistic regression was fitted to see the association between independent variables and the outcome variable after adjusting for potential confounder variables. Finally, the AOR and 95% CI was estimated and interpreted for all predictors.

### Data quality control

To maintain the quality of data all the necessary measures were done before, during and after the actual data collection. A valid and reliable instrument was adapted and used for the data collection. All data collectors and supervisors obtained a half day training on the purpose of the study, details of the data collection instrument (questionnaire), interviewing techniques, importance of privacy and ensuring confidentiality of the respondents prior to the actual data collection.

The original English version of the data collection tool was translated into Amharic and re-translated back into English to maintain its consistency. Pre-test of the tool was done at Debre Tabour district to check the understandability, consistency and appropriateness of the questionnaire.

Daily close supervision at the end of every data collection was made; the questionnaires were reviewed and checked for completeness, accuracy and consistency by supervisors and investigator to take timely corrective measures.

## Results

### Socio-demographic characteristics of participants

A total of 836 patients interviewed with a response rate of 98.8%. Majority of them 440 (52.9%) aged between 19–39 years and male participants predominated. Of the participants, 807(96.53%) are Christians. Two third of patients were rural residents (Table 1).

**Table 1:** Socio-demographic characteristics of participants in Mecha district, 2021

Variables	Categories	Frequency	Percent (%)
Age	0–18	116	13.9
	19–39	440	52.6
	40–59	230	27.5
	> = 60	50	6.0
Sex	Male	565	67.58
	Female	271	32.42
Religion	Christian	807	96.53
	Muslim	29	3.47
Residence	Urban	202	24.16
	Rural	634	75.84
Education	Unable to read and write	354	42.34
	Able to read and write	462	57.66
Marital status	Single	209	25.03
	Married	581	69.58
	Widowed	21	2.51
	Divorced	24	2.87
Occupation	Underage	155	18.54
	Merchant	69	8.25
	Housewife	95	11.36
	Farmer	465	55.62
	Employed	52	6.22
Community Based Health Insurance	Yes	431	51.56
	No	405	48.44
Annual income	First quartile (≤ 21,000)	217	25.96
	Second quartile (21,000.1–30,000)	249	29.78
	Third quartile (30,000.1, 36,500)	163	19.50
	Fourth quartile (> 36,500)	207	24.76

### Types and cause of trauma

Perceived dislocation was the leading type of trauma (37.92%) followed by strain (37.44%). Fall down (68.3%) is the main cause of injury. Extremity 77.63% was the most commonly reported site of injury (Table 2).

**Table 2:** Type and cause of trauma of participants in Mecha district, 2021

Variables	Categories	Frequency	Percent (%)
Type of trauma	Fracture	206	24.64
	Dislocation	317	37.92
	Strain	313	37.44
Type of fracture(n = 206)	Open fracture	91	44.17
	Closed fracture	115	55.83
Site of injury	Extremity	649	77.63
	Trunk	61	7.30
	Shoulder	126	15.07
Cause of injury	Fall down and Natural deformity	571	68.30
	Road traffic accident	265	31.70
Time since injury	< 3 months	30	3.59
	3–6 months	105	12.56
	6–12 months	701	83.85

### Prevalence of traditional bone setting service utilization

The prevalence of TBS utilization was 385(46.05%) with 95% CI (42.69, 49.45). Massage was most utilized type of treatment by the traditional bone setters. The most commonly reported reason for visiting TBS was acceptance of the practice by the society. The median cost for TBS treatment was 300 (IQR 200–700) Ethiopian birr. From all TBS users, 73.47% of them were stated that they were cured from their trauma. However, 11.41% of them developed complications after receiving treatment by TBS.

From all 43 patients with complication, the most commonly reported types of complications were infection (26.56%) and mal-union (20.31%). Among patients faced complication after TBS treatment 42(97.7%/ visited the nearby hospital (Table 3).

**Table 3:** Prevalence of traditional bone setter utilization and interventions in Mecha district, 2021

Variables	Categories	Frequency	Percent (%)
Interventions made by TBS	Traditional medication	384	99.74
	Referred to Hospital	1	0.26
Type of intervention	Massage	218	56.62
	Bambo Splinting	17	4.42
	Traction	110	28.57
	Traditional medication	40	10.39
Reason to utilize TBS	Peer pressure	134	34.81
	Fast service in TBS	1	0.26
	Lack of awareness and negligence	22	5.71
	Less costly in TBS	4	1.04
	TBS more accepted in society	224	58.18
Distance from home to TBS	< 30 min	76	19.74
	30–60 min	229	59.48
	60–120 min	61	15.84
	> 120 min	19	4.94
Frequency of TBS visit (*n* = 385)	< 3	85	22.08
	> = 3	300	77.92
Outcome TBS Rx	No improvement	16	4.24
	Improved	41	10.88
	Cured	277	73.47
	Complicated	43	11.41
Type of complication (*n* = 43)	Stiffness	5	7.82
	Mal-union	13	20.31
	Shortness	2	3.12
	Non union	3	4.69
	Infection	17	26.56
	Gangrene	5	7.82
	Pain	4	6.25
	Others	15	23.43

### Health care facility related characteristics

Four hundred fifty one (53.95%) of the patients visited hospital for treatment and 42(5.50%) of the patients visited both TBS and hospital. Out of 451 patients who visited hospital, 297(60.2%) were self-referral; 189(38.3%) were due to family or peer pressure; seven (1.4%) were referred by TBS. Only 4(0.8%) of the participants had no improvement and there was no complication observed on participants who visited hospital. However, 31.22% of the participants reported that TBS as their future preference (Table 4).

**Table 4:** Health care facility related characteristics of participants in Mecha district, 2021

Variables	Categories	Frequency	Percent (%)
Distance	< = 30 min	134	27.02
	30–60 min	124	25.00
	60–120 min	211	42.54
	> 120 min	27	5.44
Reason for hospital preference	Fear of mal union from TBS	133	17.43
	Fear of stiffness	13	1.70
	Amputation	46	6.03
	Family /peer pressure	128	16.77
	Good infection prevention	10	1.32
	Prolonged healing at TBS	7	0.92
	Better health care providers competency	198	25.95
	Better outcome in hospital	228	29.88
Treatment type(*n* = 497)^a^	Medication	245	49.30
	Surgery	92	18.51
	Bandage	110	22.13
	Traction	50	10.06
Outcome of hospital treatment(*n* = 497)^a^	No improvement	4	0.80
	Improved	146	29.38
	Cured	347	69.82
	Complicated	0	0
Frequency of Hospital visit	< 3	282	56.74
	> = 3	215	43.26
Future Recommendation(n = 836)^a^	Hospital	575	68.78
	TBS	261	31.22

^a^ indicates the value includes both hospital visitors and first TBS then Hospital visitors

### Factors associated with TBS utilization

From the final model, age, residence, occupation, type of trauma, site of trauma, cause of trauma and annual income were factors significantly associated with utilizations of TBS practice.

Our study revealed that patients who are aged at least 60 years old were 87% less likely to utilize TBS than those who were under 18 years (AOR = 0.13, 95% CI: 0.03,0.43). Rural dwellers were 3.63 times more likely (AOR = 3.63, 95% CI: 1.76 – 7.50) to utilize TBS as compared to the urban dwellers keeping the other variables constant. The study also found higher odds of TBS utilization among patients who are housewife as compared to employed participants (AOR = 4.12, 95% CI: 1.33 – 12.70). However, merchants are 79% less likely to utilize TBS as compared to employed participants (AOR = 0.21, 95% CI: 0.07 – 0.61). Similarly, type of injury, site of injury, cause of trauma and annual income were significantly associated with utilizations of TBS (Table 5).

**Table 5:** Factors associated with TBS utilization in Mecha district, 2021

Variables	Categories	TBS Utilization		COR (95%CI)	AOR (95% CI)
		Yes	NO		
Age	0–18	51	65	1	1
	19–39	226	214	1.35(0.89,2.03	1.37(0.67,2.80)
	40–59	96	134	0.91(0.58,1.43)	1.05(0.44, 2.54)
	> = 60	12	38	0.40(0.19,0.84)	0.13(0.03,0.43)^a^
Sex	Male	256	309	1	1
	Female	129	142	1.09(0.82,1.36)	1.01(0.63,01.64)
Religion	Christian	371	436	1	1
	Muslim	14	15	1.10(0.52,2.30)	1.58(0.57,4.39)
Residence	Urban	62	140	1	1
	Rural	323	311	2.35(1.67,3.29)	3.63(1.76, 7.50)^a^
Occupation	Underage	66	89	2.01(1.01, 4.02)	0.77(0.28, 2.19)
	Merchant	10	59	0.46(0.19,1.14)	0.21(0.07,0.61)^a^
	Housewife	79	16	13.40(5.93, 30.27)	4.12(1.34, 12.70)^a^
	Farmer	216	249	2.35(1.24, 4.46)	1.43(0.51, 3.97)
	Employed	14	38	1	1
Types of trauma	Fracture	36	170	1	1
	Dislocation	218	99	10.40(6.76,16.00)	6.40(3.69,11.10)^a^
	Strain	131	182	3.40(2.22, 5.19)	2.09(1.05,4.14)^a^
Site of injury	Extremity	341	308	1	1
	Trunk	7	54	0.12(0.05,0.26)	0.08(0.03,0.22)^a^
	Shoulder	37	89	0.38(0.25,0.57)	0.20(0.11, 0.37)^a^
Cause of injury	Fall dawn and Natural deformity	344	227	8.27(5.70, 12,01)	9.87(5.93, 16.42)^a^
	RTA (accidents)	41	224	1	1
Annual income	First quartile (≤ 21,000)	96	121		1
	Second quartile (21,000.1–30,000)	118	131	1.13(0.78, 1.63)	1.29(0.77, 2.18)
	Third quartile (30,000.1, 36,500)	80	83	1.21(0.81, 1.82)	1.29(0.72, 2.33)
	Fourth quartile (> 36,500)	91	116	0.98(0.67, 1.45)	2.33(1.29, 4.22)^a^

^a^statistically significant at 0.05

## Discussion

Traditional bone setters are one of health care choices of communities worldwide and it enjoys enormous acceptance in developing countries including Ethiopia. As a result; we set out to determine the community prevalence of TBS users in Mecha district,Amhara Regional State, Ethiopia. Acccordingly, the study revealed that the prevalence of TBS utilization was 46.05% (95% CI: 42.69, 49.45), which relate to the study done in Nigeria [11]. Even though our study included all injuries, the result is also consistent with the study done on fractured patients in Wolaita Sodo, Ethiopia, 45.7% [2]. This indicates that TBS is one of the most important treatment choices for large number of the population.

However, it is lower than the studies done in Kano, Nigeria, which was 60.5% [14] and Northern region of Ghana, 65.7% [15]. On the other hand, it is higher than the studies done in Tanzania 6.3% [16], rural areas of Nigeria, 25.6% [17] and in Ilorin, north central Nigeria, 28.7% [9]. This difference could be due to the differences in the study setting, and socio-demographic characteristics of study participants in which Ethiopia is less developed than those listed settings.

Of those TBS users, the majority, 56.62% of the participants used to receive massage as the first intervention followed by traction, traditional medication and splinting with bamboo28.57%, 10.39% and 4.42% respectively. This finding is similar with the study done in Nigeria [18]. Major reasons for TBS preference were society acceptance (58.18%), peer pressure (34.81%), lack of awareness (5.71%) and less costly of TBS practice (1.04%), which is more or less similar with a study done in Nigeria [18]. Whereas, the major reasons to visit hospital were expectation of better outcome in the hospital (29.88%), better health care providers competency (25.95%) and fear of mal-union from TBS treatment 17.43%). From all TBS user patients, 43(11.41%) complications were observed whereas, we failed to observe any complicated case in patients who visited modern hospital.

In this study age, residence, occupation, type of injury, site of injury, cause of trauma and annual income were significantly associated with utilizations of TBS. Older aged patients were 87% less likely to use TBS than younger ones. This result is in line with study conducted in Nigeria [9]. This might be due to younger age group belongs to the productive and the working populations who are mostly involved in bone trauma due to participation in injury prone activities.

Our findings showed that patients from rural residence were 3.63 times more likely to utilize TBS than patient from urban. The plausible explanation could be increased health related literacy among urban residents than rural regarding TBS and the limited accessibility of health facilities in the rural area. This result is dissimilar with previous study done in Nigeria [14]. This difference could be attributed to study design in which the Nigerian one was conducted at institution based.

Furthermore, this study found that patients who had housewife occupation were more likely and merchants were less likely to utilize TBS practice than patients who were employed which is supported by a study done in Nigeria [9]. The possible reason is that patients who had housewife occupation have less awareness regarding TBS outcome complication and they may not have the power to decide where to go because in Ethiopian context housewives are usually less or none educated and they might be influenced by the heads of households or spouses involved in decision making in the household. However, the observation of some study showed that occupation had not been significantly associated with TBS utilization [14, 18].

In addition, we found that type of trauma was statistically associated with utilization of TBS. Patients with dislocation were 6.40 (95% CI: 3.69 – 11.10) times more likely to visit traditional bone setters than patients with fracture. Those patients with strain were 2.09 (95% CI: 1.05 – 4.14) times more likely utilized TBS than patients with fracture. The probable justification might be the perception of the community that fracture is more severe and related with more complication than strain and dislocation. Patients with trunk injury were 91% less likely and individuals with shoulder injury were 80% less likely to use traditional bone setting practice as compared with individuals who had trauma at the extremity. This finding was consistent with the studies done in Kano and rural area of Nigeria [14, 18]. This might be due to the understanding of the community, problems in the extremity are less severe than trunk injuries, and extremity injuries can be managed easily by TBS practitioners.

Regarding the cause of trauma, patients with fall down injury and natural deformity were 9.87 times more likely to use TBS than those with other accidents which are reinforced by the study done in Wolaita Sodo, Ethiopia [3]. The reason behind might be accidents including RTA, bullet and stick injury could cause life threatening bleeding and have medico-legal issues that forces to visit hospitals.

Income was one of the significantly associated factors in this study. Patients with household annual income greater than 36,500 birr were 2.34 times more likely to utilize TBS compared to those who had less than 21,000 birr per year which is supported by a previous study [9]. There are also studies contradicted it [19, 20]. In this study only 1.04% of TBS users mentioned low cost as a reason to prefer TBS than modern care.

The strength of this study is its usage of representative community based primary data. On the other hand, it has limitations which relate possible recall bias and the cross-sectional nature of the study which would not allow us to establish causal relationship.

## Conclusions

The prevalence of traditional bone setting utilization is high in the study area, despite recent advancements in the practice of orthopedics and trauma care in Ethiopia. Rural residence, housewife occupation, dislocation and strain type of trauma, trauma of extremity, fall down and natural deformity and high annual income were factors which favors utilize of traditional bone setting services. Since TBS services are more accepted in the society, the integration of TBS into the health care delivery system is recommended to prevent complications from TBS malpractices.

## Data Availability

The datasets used and/or analyzed during the current study are available from the corresponding author on reasonable request.
